# Trends and factors associated with repeated adolescent pregnancies in Tanzania from 2004-2016: evidence from Tanzania demographic and health surveys

**DOI:** 10.11604/pamj.2021.40.162.29021

**Published:** 2021-11-17

**Authors:** Octavian Aron Ngoda, Innocent Baltazar Mboya, Michael Johnson Mahande, Sia Emmanuel Msuya, Jenny Renju

**Affiliations:** 1 Department of Epidemiology and Biostatistics, Institute of Public Health, Kilimanjaro Christian Medical University College (KCMUCo), Moshi, Tanzania,; 2 School of Mathematics, Statistics and Computer Science, University of Kwazulu-Natal, Pietermaritzburg, Scottsville, South Africa,; 3 Department of Community Medicine, Institute of Public Health, Kilimanjaro Christian Medical Center (KCMC), Moshi, Tanzania,; 4 Department of Population Health, London School of Hygiene and Tropical Medicine, London, United Kingdom

**Keywords:** Repeated adolescent pregnancy, prevalence, risk factors, trends

## Abstract

**Introduction:**

a repeated pregnancy represents a failure of health and social systems to educate and provide the necessary services and skills to ensure adolescent girls do not experience any further unwanted pregnancies during this young age. We aimed to determine trends and factors associated with repeated adolescent pregnancies in Tanzania 2004-2016.

**Methods:**

an analytical cross-sectional study was conducted using secondary data from Tanzania demographic and health surveys of the years 2004-2005, 2010 and 2015-2016 among adolescent mothers aged 15 to 19 years. Data analysis was performed using STATA version 15 and considered the complex survey design. The Poisson regression model was used to estimate prevalence ratios (PR) and 95% confidence intervals for factors associated with repeated adolescent pregnancy.

**Results:**

the proportion of repeated adolescent pregnancies increased from 15.8% in 2004/2005 to 18.6% in 2010, then to 18.8% in 2015/2016. Adolescents who delivered their first pregnancy at home (APR: 1.36, 95% CI: 1.03, 1.78) and who started sexual activity before 15 years of age (APR: 1.80, 95% CI: 1.40, 2.31) were likely repeated adolescent pregnancy. In contrast, adolescents who used contraception (APR: 0.52, 95% CI: 0.34, 0.81) had a lower prevalence of repeated adolescent pregnancies.

**Conclusion:**

the prevalence of repeated adolescent pregnancies has increased and remains unacceptably high. Adolescents who had low education delivered their first pregnancy at home and were non-contraceptive users need to be targeted in policies and programs for the prevention of repeated adolescent pregnancies.

## Introduction

Globally, pregnancy during adolescence (10-19 years) is a major contributor to maternal and child morbidity and mortality [1,2]. A second pregnancy during adolescence has been associated with an increased risk of preterm birth, low birth weight, stillbirths, perinatal and neonatal mortality above and beyond the first pregnancy [3,4]. With the increased likelihood of further economic difficulties, depression and behavior disorder [5,6] and resulting in children with poorer health and educational outcomes [7,8].

The prevalence and trends of repeated adolescent pregnancies vary around the world. In Thailand and Brazil, 20.0% and 53.5% respectively of adolescents have been reported to have experienced two or more pregnancies before the age of 19 years [9,10]. In the Philippines, the prevalence of repeated adolescent pregnancy declined from 20.4% in 1993 to 18.1% in 2013 [11]. However, in Africa, there is limited data. In South Africa, two studies reported the prevalence ranged from 17.6% to 19.9% [12,13], and in Uganda the prevalence decreased from 66.8% in 2006 to 55.6% in 2016 [14].

Different factors have been reported to be associated with the occurrence of repeated adolescent pregnancy which includes: young age at first birth, low income, low education level and low use of contraceptives [4,10], inadequate knowledge about contraception, history of abortion and depression [6,15], and history of spontaneous abortion [3,12].

In Tanzania, there are various interventions aimed to reduce adolescent pregnancies including repeated adolescent pregnancies, which fall under the adolescent-friendly sexual and reproductive health (AFSRH) services. AFSRH services include health education on child spacing during antenatal care and postnatal care, family planning counseling, free provision of contraceptives at reproductive and child health clinics at all levels of health facilities [16,17]. The Ministry of Health Community Development Gender, Elderly and Children (MoHGCDEC) have set a target to reach 80% of the adolescent population with AFSRH and reducing the adolescent fertility rate to less than 90 births per 1000 adolescents by 2020 [17]. However, data from 2017 suggests that the coverage of AFSRH services is still suboptimal reaching only 63% and the 2016 adolescent fertility rates were still unacceptably high at 132 births per 1000 adolescents [17].

Accurate and timely information about the trends and factors associated with repeated adolescent pregnancy is needed to support evidence-based strategies to address the problem. In this study, we aimed to determine the trends and factors associated with repeated adolescent pregnancies in Tanzania from 2004-2016.

## Methods

**Study design and setting:** we conducted an analytical cross-sectional study using nationally representative secondary data from three Tanzanian demographic and health survey (TDHS) from 2004/2005, 2010 and 2015/2016. The study included data from all 31 regions of the United Republic of Tanzania. Tanzania has a population is 44.9 million and 23% are adolescents (~ 9.9 million) (10-19 years) [18]. The average fertility rate for women in Tanzania in 2016 was 4.71 children and women´s mean age at first birth was 19.8 years old [19].

**Study population, sample size, and sampling:** the TDHS used a multi-stage sampling technique in selecting study participants, which was designed to provide estimates for the Mainland and Zanzibar. Sampling was conducted in stages: stage one involved a selection of a stratified sample of clusters from a list of enumeration areas (EAs) that were obtained from the most recent census conducted in Tanzania, stratified by rural and urban settings. In rural areas, an EA is a natural village, or a segment of a large village, or a group of small villages; in urban areas, and EA is a street or a city block. This sample of EAs was selected using probability proportional to size (PPS) to account for the different sizes of the enumeration area. A listing procedure was then performed on each of the selected EAs such that all dwellings and households are listed. In the second stage, a fixed variable number of households were selected from a complete list of households in each of the selected EAs, using an equal probability systematic sampling technique. In each of the selected households, women aged 15-49 years were identified and then interviewed.

**Figure 1:**
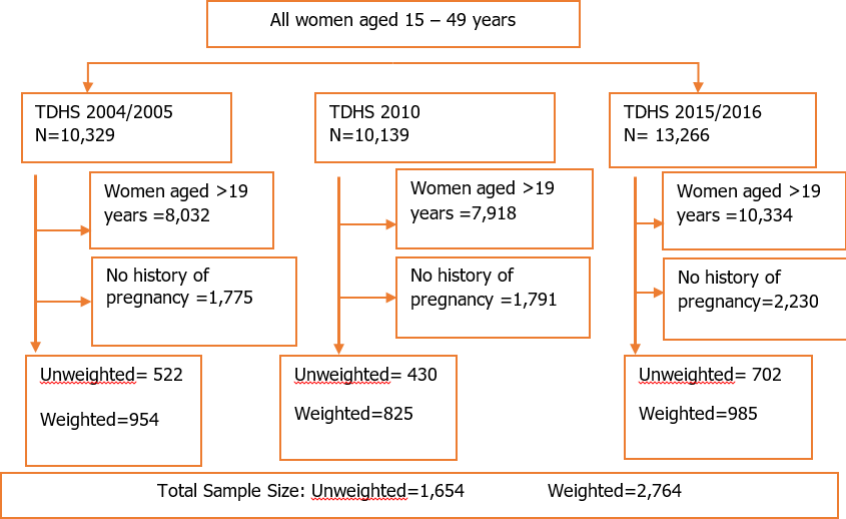
flow chart showing the sample size used in the analysis; data from the Tanzania and demographic health surveys 2004/2005-2015/2016

The study participants were adolescent girls aged 15-19 years who reported having given birth or were pregnant at the time of the interview. This resulted in a total weighted sample of 2,764 from the three TDHS; 522 adolescents in 2004/2005 (954 weighted cases), 430 adolescents in 2010 (825 weighted cases) and 702 adolescents in 2015/2016 (985 weighted cases) (Figure 1).

**Study variables and variable measurements:** the dependent variable was a repeated adolescent pregnancy. This was a binary outcome variable that coded as 1 “yes” if an adolescent girl had had at least two births or had one birth and was pregnant during the interview and 0 “no” if an adolescent girl had only had one birth or had no history of any birth but was pregnant during the interview. The study did not include pregnancies that had resulted in abortions or miscarriages owing to inconsistencies in reporting over the different rounds of the TDHS. The independent variables included socio-demographic and sexual and reproductive health characteristics. Socio-demographic characteristics included age in years (15-17, 18-19), working status (working, not working), education level (no education, primary, secondary and above), wealth index categories (poorest, poorer, middle, richer, richest), marital status (never in a union, married/cohabiting, widowed/divorced/separated), place of residence (urban-rural), geographical zone (western, northern, central, southern highlands, southern, south-west highlands, lake, eastern, Zanzibar), partner´s education level (no education, primary, secondary and above) and survey year (2004/05, 2010, 2015/16). Reproductive health characteristics included age at first sex in years (<15, ≥15), age at first marriage in years (<15, 15-17, 18-19), age at first birth in years (<15, 15-17, 18-19), number of ANC visits at first pregnancy (<4, ≥4), place of delivery at first pregnancy (home, health facility) and modern contraceptive use (no, yes).

**Statistical analysis:** data analysis was performed using STATA version 15 and accounted for the complex nature of survey design through the application of weights, primary sampling unit (cluster) and strata for the adjustment of the cluster sampling survey design. Descriptive statistics were summarized using frequency and proportions with standard deviations for categorical variables and continuous variables using mean and median with interquartile ranges (IQR). Poisson regression analysis was used to determine factors associated with repeated adolescent pregnancy as an alternative to the classical logistic regression given that the proportion of repeated adolescent pregnancies was greater than 10%. The choice of this model was also motivated by the non-convergence of the log-binomial regression model. Bivariate Poisson regression was conducted to examine the unadjusted association between exposure variables and the likelihood of repeated adolescent pregnancies. Variables with p-value<0.05 in the bivariate analysis were entered in the multivariable model to adjust for the potential confounding effect. We used stepwise forward elimination regression methods for model building. Models with the lowest Akaike information criteria (AIC) were regarded as parsimonious. Multicollinearity was assessed among the explanatory variables to be included in the Poisson regression model by inspecting the correlation matrix and assessing estimation problems of the model parameters.

**Ethical considerations:** ethical approval was obtained from the Kilimanjaro Christian Medical University College Research and Ethics Review Committee (CRERC no.PG/012/2019). The parent study obtained written, informed consent from study participants. Interviews were conducted in a private place around the household, and participants identified using unique identification numbers to ensure confidentiality and privacy of participant information. Permission to download and use the DHS data was granted from the DHS program. Data was used solely for the current study.

## Results

**Socio-demographic characteristics of study participants:** a total of 2764 adolescents reported having had at least one pregnancy. Most of the participants were from the rural areas; 739 (77.5%), 669 (81.1%), and 731 (74.2%) for 2004/05, 2010, and 2015/16 surveys respectively. The mean age (±SD) of the study population was 17.9 (±1.1) for 2004/05, 17.8 (±1.2) for 2010, 18.0 (±1.0) years for 2015/16 survey. More than half of the respondents in each survey had at least a primary education level. The majority of the participants, 682 (71.4%), 534 (64.7%) and 643 (65.2%) for the 2004/05, 2010 and 2015/16 survey respectively were either married or cohabiting (Table 1).

**Table 1:** socio-demographic characteristics (weighted) of study participants in TDHS 2004/2005, 2010 and 2015/2016

	Survey year		
Variable	2004/05 (n=954) n (%)	2010 (n=825) n (%)	2015/16 (n=985) n (%)
**Women age (years)**			
15-17	281 (29.4)	287 (34.7)	264 (26.8)
18-19	673 (70.6)	538 (65.3)	721 (73.2)
Mean ± SD	17.9 ± 1.1	17.8 ± 1.2	18.0 ± 1.0
Working status *			
Not working	202 (21.3)	196 (24.0)	374 (37.9)
Working	748 (78.7)	621 (76.0)	611 (62.1)
**Women education level**			
No education	325 (34.0)	156 (18.9)	116 (11.7)
Primary	612 (64.2)	594 (72.1)	734 (74.6)
Secondary and above	17 (17.8)	75 (9.0)	135 (13.7)
**Wealth index**			
Poorest	186 (19.5)	124 (15.1)	271 (27.5)
Poorer	239 (25.0)	197 (23.8)	225 (22.9)
Middle	192 (20.2)	198 (24.0)	168 (17.1)
Richer	170 (17.9)	181 (22.0)	182 (18.4)
Richest	167 (17.4)	125 (15.1)	139 (14.1)
**Marital status**			
Never in union	224 (23.5)	252 (30.5)	273 (27.7)
Married/cohabiting	682 (71.4)	534 (64.7)	643 (65.2)
Widowed/divorced/separated	48 (05.1)	39 (04.7)	69 (07.1)
**Place of residence**			
Urban	215 (22.5)	156 (18.9)	254 (25.8)
Rural	739 (77.5)	669 (81.1)	731 (74.2)
**Geographical zones**			
Western	123 (12.8)	83 (10.0)	157 (16.0)
Northern	73 (7.6)	69 (8.4)	66 (6.7)
Central	85 (9.0)	71 (8.6)	107 (10.8)
Southern highlands	62 (6.5)	39 (4.7)	49 (4.9)
Southern	65 (6.9)	43 (5.2)	40 (4.1)
Southwest	98 (10.3)	81 (9.8)	115 (11.7)
Lake zone	339 (35.5)	333 (40.4)	315 (32.0)
Eastern	97 (10.2)	98 (11.9)	126 (12.9)
Zanzibar	12 (1.2)	8 (1.0)	10 (1.0)
Partner education level *			
No education	157 (21.6)	110 (19.3)	80 (12.5)
Primary	525 (72.0)	419 (73.5)	432 (67.3)
Secondary and above	47 (6.4)	41 (7.2)	130 (20.2)

* Information for working status was 950, 817 participants for 2004/05 and 2010 respectively partner education level was 729, 570 and 642 participants for 2004/05, 2010 and 2015/16 survey years respectively

**Sexual and reproductive characteristics of the study population:** the median age (IQR) at first sexual intercourse was 15.0 (2) and age at first birth was 17.0 (2 years for all the survey years). Nearly one in four adolescents in the three surveys (24.0% - 29.0%) reported starting sex at < 15 years. Modern contraceptive use increased from 10.6% in the 2004/05 survey to 17.9% in the 2015/16 survey (Table 2).

**Table 2:** sexual and reproductive health (SRH) history (weighted) of study participants in TDHS 2004/2005, 2010 and 2015/2016

	Survey year		
Variable	2004/05 (n=954) n (%)	2010 (n=825) n (%)	2015/16 (n=985) n (%)
Age at first sex (years) *			
<15	180 (24.0)	165 (29.0)	276 (28.1)
≥15	572 (76.0)	404 (71.0)	708 (71.9)
Median (IQR)	15 (2)	15 (2)	15 (2)
Age at first marriage (years) *			
<15	133 (18.2)	90 (15.7)	105 (14.7)
15-17	501 (68.6)	384 (67.0)	485 (68.2)
18-19	96 (13.2)	99 (17.3)	122 (17.1)
Median (IQR)	15 (2)	15 (2)	15 (2)
Age at first birth *			
<15	39 (5.5)	37 (5.9)	25 (3.3)
15-17	469 (65.4)	429 (69.2)	484 (62.5)
18-19	209 (29.1)	154 (24.9)	265 (34.2)
Median (IQR)	16 (2)	16 (2)	16 (2)
Modern contraceptive use *			
No	828 (89.4)	686 (85.3)	801 (82.1)
Yes	98 (10.6)	118 (14.7)	175 (17.9)
Place of delivery at first pregnancy *			
Home	316 (44.3)	245 (40.0)	217 (28.4)
Health facility	397 (55.7)	367 (60.0)	546 (71.6)
Number of ANC Visits at first pregnancy *			
<4	269 (37.8)	394 (63.8)	404 (52.6)
≥4	442 (62.2)	224 (36.2)	365 (47.4)

* Information for age at first sex was 752, 749 and 984 participants, for age at first marriage was 730, 573 and 712 participants, for age at first birth was 717, 620 and 774 participants, for modern contraceptive use was 929, 804 and 976 participants, for place of delivery was 713, 612, 763 participants and for number of ANC visit was 711, 618 and 769 participants for 2004/05, 2010 and 2015/16 survey years respectively

**Figure 2:**
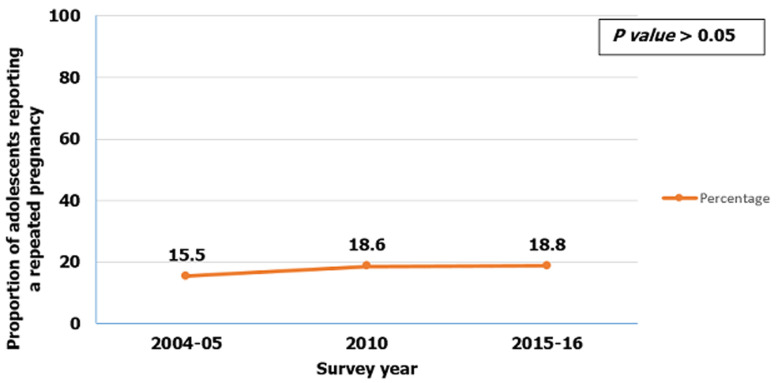
trends of repeated adolescent pregnancy in ten years; data from Tanzania and demographic health surveys 2004/2005-2015/2016

**Figure 3:**
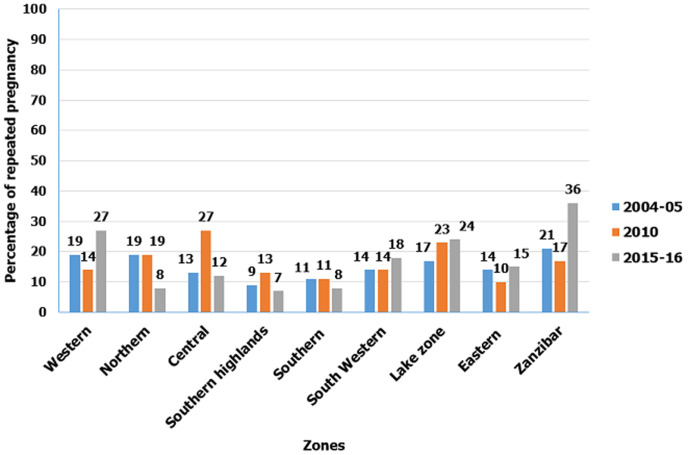
prevalence of repeated adolescent pregnancy by zones in the ten years. Data from the Tanzania and demographic health surveys 2004/2005-2015/2016

**Trends of repeated adolescent pregnancies from 2004 to 2016:** the proportion of repeated adolescent pregnancies increased from 15.8% in 2004/5 to 18.6% in 2010, and then to 18.8% in 2015/2016, this increase was not statistically significant (p-value>0.05) (Figure 2). Repeated adolescent pregnancies increased in central (13% to 27%), southern highlands (9% to 13%), and Lake zone (7% to 23%) from the survey year 2004/2005 to 2010, yet remained constant in Northern, Southern, and South-Western regions over the same period. Between 2010 and 2015/2016 survey years the prevalence of repeated adolescent pregnancies increased in Western (14% to 27%), South-Western (14% to 18%), Lake (23% to 24%), Eastern (10% to 15%) and Zanzibar (17% to 36%) zones, in the survey year 2015/2016 (Figure 3).

**Factors associated with repeated adolescent pregnancies between 2004/2005 - 2015/2016:** in the bivariate Poisson regression analysis, the following independent variables were significantly associated with repeated adolescent pregnancies: adolescent age, women´s education level, household wealth index, marital status, geographical zones, place of residence, place of delivery at first pregnancy, age at first birth, number of antenatal care (ANC) visit at first pregnancy, age at first sexual activity and modern contraceptive use. In the multivariable analysis adolescent age, geographical zones, place of delivery at first pregnancy, education level, marital status, modern contraceptive use and age at first sexual activity remained independently associated with repeated adolescent pregnancy after adjusting for other variables (Table 3). Adolescents aged 18-19 years had a 2.37 (APR: 2.37, 95% CI: 1.58, 3.55) higher prevalence of repeated pregnancies compared to those aged 15-17 years. The prevalence of repeated pregnancy was 2.23 (APR: 2.23, 95% CI: 1.42, 3.50) and 1.96 (APR: 1.96, 95% CI: 1.07, 3.60) times higher among married/cohabiting and widowed/divorced/separated adolescents respectively compared to those never in a union. The prevalence of repeated adolescent pregnancy was 2.29 (APR: 2.29, 95% CI: 1.37, 3.86) times higher among adolescents living in Zanzibar compared to those living in western Tanzania mainland. Adolescents who started sexual activity at age <15 years old had an 80% (APR: 1.80, 95% CI: 1.40, 2.31) higher prevalence of repeated pregnancies compared to those starting at age ≥15 years. The prevalence of repeated pregnancies was 36% (APR: 1.36, 95% CI: 1.03, 1.78) higher among adolescents who delivered at home compared to those who delivered at health facilities during their first pregnancies. Adolescents girls with secondary education and above had a 78% (APR: 0.22, 95% CI: 0.09, 0.51) lower prevalence of repeated pregnancy compared to those with no education. The prevalence of repeated pregnancies was 48% (APR: 0.52, 95% CI: 0.34, 0.81) lower among contraceptive users compared to non-contraceptive users (Table 3).

**Table 3:** factors associated with repeated adolescent pregnancies in Tanzania, 2004/2005 to 2015/2016

Variables	Total	Repeated n (%)	CPR* (95%CI)	p-value	APR** (95% CI)	p-value
**Women age**						
15-17	832	64 (7.7)	1		1	
18-19	1932	424 (22.0)	2.87 (1.90, 4.35)	<0.001	2.37 (1.58, 3.55)	<0.001
**Women education level**						
No education	596	159 (26.7)	1		1	
Primary	1942	322 (16.6)	0.48 (0.39, 0.80)	<0.001	0.78 (0.58, 1.04)	0.064
≥ secondary	226	7 (3.1)	0.12 (0.06, 0.25)	<0.001	0.22 (0.09, 0.51)	<0.001
**Household wealth index**						
Poorest	581	145 (25.0)	1		1	
Poorer	661	135 (20.4)	0.82 (0.59, 1.13)	0.217	1.11 (0.55, 2.26)	0.766
Middle	558	89 (15.9)	0.64 (0.45, 0.90)	0.011	1.29 (0.65, 2.60)	0.464
Richer	533	77 (14.4)	0.58 (0.39, 0.85)	0.006	1.07 (0.52, 2.19)	0.855
Richest	431	42 (9.8)	0.39 (0.24, 0.63)	<0.001	1.15 (0.58, 2.29)	0.690
**Marital status**						
Never in union	749	48 (6.4)	1		1	
Married/cohabiting	1858	408 (22.0)	3.44 (2.07, 5.72)	<0.001	2.23 (1.42, 3.50)	<0.001
Widowed/divorced/ separated	157	32 (20.1)	3.14 (1.63, 6.00)	0.001	1.96 (1.07, 3.60)	0.030
**Place of residence**						
Urban	625	65 (10.4)	1		1	
Rural	2139	422 (19.8)	1.89 (1.32, 2.71)	<0.001	1.02 (0.64, 1.62)	0.932
**Geographical zones**						
Western	362	78 (21.5)	1		1	
Northern	208	32 (15.6)	0.73 (0.40, 1.30)	0.282	1.22 (0.67, 2.23)	0.516
Central	264	43 (16.3)	0.76 (0.46, 1.26)	0.285	0.88 (0.53, 1.46)	0.629
Southern highlands	149	14 (9.2)	0.43 (0.21, 0.86)	0.017	0.64 (0.28, 1.48)	0.296
Southern	148	14 (10.0)	0.47 (0.26, 0.84)	0.012	0.64 (0.34, 1.23)	0.183
South west	294	45 (15.6)	0.72 (0.46, 1.14)	0.157	1.21 (0.71, 2.08)	0.485
Lake zone	988	210 (21.4)	0.99 (0.71, 1.38)	0.970	1.18 (0.82, 1.68)	0.374
Eastern	322	42 (13.2)	0.61 (0.36, 1.03)	0.064	1.10 (0.63, 1.90)	0.737
Zanzibar	29	7 (24.9)	1.16 (0.76, 1.75)	0.493	2.29 (1.37, 3.86)	0.002
**Modern contraceptive use**						
No	2315	435 (18.8)	1		1	
Yes	449	53 (11.8)	0.63 (0.43, 0.92)	0.016	0.52 (0.34, 0.81)	0.004
**Age at first sex**						
<15	622	168 (27.0)	1.97 (1.53, 2.55)	<0.001	1.80 (1.40, 2.31)	<0.001
≥15	1684	230 (13.7)	1		1	
**Place of delivery at first pregnancy**						
Health facility	1311	217 (16.6)	1		1	
Home	776	267 (34.4)	2.07 (1.67, 2.56)	<0.001	1.36 (1.03, 1.78)	0.028
**Number of ANC visits at first pregnancy**						
<4	1068	299 (28.0)	1.54 (1.21, 1.96)	<0.001	1.28 (0.99, 1.64)	0.057
≥4	1031	187 (18.2)	1		1	
**Age at first birth**						
<15	101	48 (48.2)	1			
15-17	1383	389 (28.1)	0.58 (0.43, 0.79)	0.001		
18-19	627	50 (8.0)	0.17 (0.10, 0.27)	<0.001		

* CPR: crude prevalence ratio: **APR: adjusted prevalence ratio: ANC antenatal care

## Discussion

The study aimed to determine the prevalence, trends and factors associated with repeated adolescent pregnancies in Tanzania from 2004 to 2016. We found increased trends in repeated adolescent pregnancies from 15.5% in 2004/2005 to 18.6% in 2010 and to 18.8% in 2015/2016, though this increase was not statistically significant. The highest proportion of repeated adolescent pregnancy was in Zanzibar, Lake, and Western zones. Education level, marital status, age at first sexual intercourse, zones, adolescent age, modern contraceptive use, and place of delivery at first pregnancy were independently associated with repeated adolescent pregnancies.

The present study demonstrated that repeated adolescent pregnancy in Tanzania had increased from 2004 to 2016. The findings contrast with the studies in Uganda and the Philippines [11,14]. These findings might suggest that interventions such as those that aimed to increase family planning use have not adequately prevented or delayed the occurrence of repeated pregnancies among adolescents who had already given birth. In our study we found that modern contraceptive use was low ranging from 10% to 17%, despite the commitments to improve family planning programs in the country [17]. Whilst the initial adolescent health policy in Tanzania aimed to reduce unwanted pregnancies and provide adolescent-friendly health services, it did not include strategies for dealing with the prevention of secondary pregnancies [17]. Further efforts are needed to specifically target adolescents who have already given birth.

In addition to family planning, our study findings support the drive to keep ensure all adolescents stay in school. Adolescent mothers with secondary education and above had a lower prevalence of repeated pregnancies compared to those with no education. Our findings are similar to previous studies in South Africa and Brazil [4,10,13,16]. Policies and strategies that mandate the expulsion of pregnant schoolgirls and limit their continued access to education are likely to further contribute to the increase in adolescent fertility rates. Additionally, keeping adolescents in school has been shown to increase the age of sexual debut [20].

In this study, we found that adolescents who started sexual activity before 15 years of age had a higher prevalence of repeated adolescent pregnancies compared to those who started at an age greater or equal to 15 years. This could be attributed to adolescents who started sexual activity before 15 years of age got first pregnancy at a younger age allows a greater amount of time of exposure to repeat pregnancy risk during teenage years, as also reported in a study conducted in Brazil [4]. Education has been widely reported to enable adolescents to become more autonomous, more knowledgeable about health care services, thus able to make healthy reproductive choices [13]. Keeping girls in school longer delays the onset of sexual activity and can therefore serve to reduce the occurrence of repeated adolescent pregnancies. Continued efforts are needed to ensure access to education for all adolescents.

Studies have reported the challenges in the implementation of truly AFSRH services and more specifically with effectively moderating health worker attitudes and the ability to provide adolescents with contraception [17]. Our study did find that interaction with the formal health service (in the form of previous delivery at a facility) did reduce the likelihood of repeated pregnancy, suggesting that post-delivery care provided by health care workers, perhaps with the inclusion of counseling on contraceptive use and child spacing could have positive implications on maternal health. Our findings call for strengthened effort to ensure that every contact between healthcare workers and pregnant adolescents be utilized as an opportunity to prevent repeat adolescent pregnancy. In our study we still found that adolescents who delivered outside of the health facility, we, therefore, recommend that additional qualitative research should also be conducted to explore the reasons adolescents have for choosing not to deliver in a health facility.

Finally, we found that married or cohabiting adolescent mothers and those who were widowed/divorced/separated had a higher prevalence of repeated adolescent pregnancy respectively compared to those who were never in a union. This could be possible because married/cohabited adolescents are expected to procreate and perhaps less able to negotiate contraception use [16]. In Tanzania the legal age of marriage is 15 years for girls. Ultimately meaning girls who marry young are at increased risk of pregnancy and repeated pregnancies. Further advocacy is needed to change the legal age of marriage and to promote a young women´s agency in her own reproductive choices.

The study utilized nationally representative data, which allows for wider generalizability of the findings. This study has estimated trends and factors associated with adolescent pregnancies in Tanzania, these findings are critical to inform policy decision-makers. There are a number of limitations that should be considered when interpreting our findings. Age at first sex data was self-reported by adolescent girls, which might have led to recall bias and an over-estimation or under-estimation of the effect. Furthermore, being cross-sectional, we were not able to establish causal relationships. Also, our analysis was limited to looking at births and current pregnancies and does not capture adolescent pregnancies that end in miscarriage or abortion. Context and characteristics could be different for adolescent pregnancies ending in miscarriage or abortion.

## Conclusion

There is a steadily increasing trend of repeated adolescent pregnancy in Tanzania from 2004 to 2016. These findings indicate the need for secondary prevention programs that encourage marriage later, ensuring facility-based delivery, support to remain in formal education and efforts to promote postpartum contraception use. We suggest qualitative research to investigate reasons and motivators for repeated adolescent pregnancies for adolescents, partners and their parents. Furthermore, we suggest qualitative research into the reasons why adolescents are not delivering in health systems.

**Funding:** this study was supported by Welcome Trust through the sub-Saharan African Consortium of Applied Biostatistics (SSACAB). The funders did not have any role or influence in the design of the study, data collection, analysis, or interpretation of the results and the development of the manuscript.

### What is known about this topic

- Pregnancy and childbirth during adolescence significantly contribute to increased maternal and child morbidity and mortality;
- A repeated pregnancy represents a failure of health and social systems to educate and provide the necessary services and skills to ensure adolescent girls do not experience any further unwanted pregnancies during this young age.

### What this study adds

- This study found the increasing trend of repeated adolescent pregnancy in Tanzania from 2004 to 2016;
- Support the need for secondary prevention programs that encourage marriage later, ensuring facility-based delivery, support to remain in formal education and efforts to promote postpartum contraception use to prevent repeated adolescent pregnancies.

## References

[1] United Nations Fund for Population Activities (UNFPA) (2015). Girlhood, not motherhood: preventing adolescent pregnancy.

[2] World Health Organization (WHO) (2020). Adolescent pregnancy fact sheets.

[3] Akinbami LJ, Schoendorf KC, Kiely JL (2000). Risk of preterm birth in multiparous teenagers. Arch Pediatr Adolesc Med.

[4] Galvão RB, Figueira CO, Borovac-Pinheiro A, Paulino DS, Faria-Schützer DB, Surita FG (2018). Hazards of repeat pregnancy during adolescence: a case-control study. Rev Bras Ginecol Obstet.

[5] Corcoran J (2016). Teenage pregnancy and mental health. Societies.

[6] Maravilla JC, Betts KS, Couto E Cruz C, Alati R (2017). Factor´s influencing repeated teenage pregnancy: a review and meta-analysis. Am J Obstet Gynecol.

[7] World Health Organization (2004). Adolescent pregnancy: issues in adolescent health and development.

[8] Cook SM, Cameron ST (2020). Social issues of teenage pregnancy. Obstetrics, Gynaecology and Reproductive Medicine.

[9] Talungchit P, Lertbunnaphong T, Russameecharoen K (2017). Prevalence of repeat pregnancy including pregnancy outcome of teenage women. Siriraj Medical Journal.

[10] Zanchi M, Mendoza-Sassi RA, Silva MR, Almeida SG, Teixeira LO, Gonçalves CV (2017). Pregnancy recurrence in adolescents in Southern Brazil. Rev Assoc Med Bras 1992.

[11] Maravilla JC, Betts KS, Alati R (2018). Trends in repeated pregnancy among adolescents in the Philippines from 1993 to 2013. Reprod Health.

[12] Mphatswe W, Maise H, Sebitloane M (2016). Prevalence of repeat pregnancies and associated factors among teenagers in KwaZulu-Natal, South Africa. Int J Gynaecol Obstet.

[13] Govender D, Naidoo S, Taylor M (2019). Prevalence and risk factors of repeat pregnancy among South African adolescent females. Afr J Reprod Health.

[14] Amongin D, Nakimuli A, Hanson C, Nakafeero M, Kaharuza F, Atuyambe L (2020). Time trends in and factors associated with repeat adolescent birth in Uganda: analysis of six demographic and health surveys. PloS One.

[15] Albuquerque AP, Pitangui AC, Rodrigues PM, Araújo RC (2017). Prevalence of rapid repeat pregnancy and associated factors in adolescents in Caruaru, Pernambuco. Revista Brasileira de Saúde Materno Infantil.

[16] Ministry of Health and Social Welfare, Tanzania (2013). National family planning guidelines and standards.

[17] Ministry of Health Community Development Gender Elderly and Children, United Republic of Tanzania (2016). One plan II: the national road map strategic plan to improve reproductive, maternal, newborn, child & adolescent health in Tanzania (2016-2020).

[18] Tanzania National Bureau of Statistics (2019). Tanzania in figures 2018.

[19] Ministry of Health, Community Development, Gender Elderly and Children (MoHCDGEC) (Tanzania Mainland), Ministry of Health (MoH) (Zanzibar), National Bureau of Statistics (NBS), Office of the Chief Government Statistician (OCGS) (2016). Tanzania demographic and health survey and malaria indicator survey (TDHS- MIS) 2015-16.

[20] Ministry of Health, Community Development, Gender, Elderly and Children (Tanzania) (2017). National survey on the drivers and consequences of child marriage in Tanzania. Girles Not Brides.

